# Early detection of velamentous cord insertion at the eighth week of gestation

**DOI:** 10.1002/ccr3.2815

**Published:** 2020-04-20

**Authors:** Caiyun Liao, Irene Peregrin‐Alvarez, Robert Roman, Joshua Morris, Laura Detti

**Affiliations:** ^1^ Department of Obstetrics and Gynecology University of Tennessee Health Science Center Memphis Tennessee; ^2^ Women’s Health Institute Department of Subspecialty Women’s Health Cleveland Clinic Cleveland Ohio

**Keywords:** assisted reproductive technology, early diagnosis, ultrasound, velamentous cord insertion

## Abstract

Velamentous cord insertion can be diagnosed at 8 weeks of gestation, earlier than previously reported. Fetal surveillance may be informed and prognosis may be impacted by early diagnosis once viability is reached.

## Graphical abstract

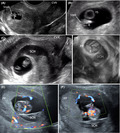

## 1 INTRODUCTION

The placenta cord insertion (PCI) site can be described as central, eccentric, marginal, and velamentous (membranous).^1^ Among PCIs, greater than 90% were central or eccentric.^1^ Marginal cord insertion (MCI) is generally defined as PCI within 1‐2 cm of the placental edge in late gestation. In velamentous cord insertion (VCI), the umbilical vessel inserts into the membrane, traverses between the amnion and chorion, and is unprotected by the Wharton's jelly or the placenta chorionic plate.^1^ If the velamentous vessels are located at the lower uterine segment, these vessels may be prone to compression and rupture at the time of labor and delivery, which may lead to rapid fetal exsanguination. VCI is also associated with preterm prelabor rupture of membranes, spontaneous preterm birth, small for gestational age, low birthweight, short cord, and emergency cesarean deliveries.^1^, ^2^ In a population study based on the Medical Birth Registry of Norway, MCI and VCI were observed in 6.3% and 1.5% of the pregnancies, respectively.^3^ Although both MCI and VCI are considered abnormal, VCI was associated with more pregnancy complications compared with MCI.^2^ Studies published on VCI to date have been largely based on ultrasound imaging in the second trimester or beyond, except in very few cases, where VCIs were diagnosed in the first trimester. Sepulveda reported diagnoses of five cases of VCIs between 11 and 14 weeks (median 12 weeks, earliest 11 + 1/7 weeks), and Hasegawa et al reported diagnosis of VCI at eight weeks of gestation in a woman with vanishing twin, who conceived through in vitro fertilization (IVF) plus intracytoplasmic sperm injection (ICSI).^4^, ^5^ Here, we report a case of VCI first detected at eight weeks of gestation with ultrasound, in a woman with a singleton pregnancy conceived with IVF plus ICSI. Informed consent was obtained from the patient.

A 32‐year‐old nulligravida African American female presented with a two‐year history of chronic pelvic pain, stage IV endometriosis, and infertility. She also had a prior history of bilateral tubal‐ovarian abscesses after a hysterosalpingogram performed at an outside institute. This was treated with ultrasound‐guided drainage and antibiotics. On her initial evaluation by us, sonohysterogram revealed bilateral hydrosalpinx, a 2 cm anterior intramural‐subserosal fibroid, and a 2 cm fundal‐posterior, intramural fibroid. The uterine cavity appeared normal. In light of hydrosalpinx, she underwent laparoscopic bilateral salpingectomy, followed by IVF.

**Figure 1:**
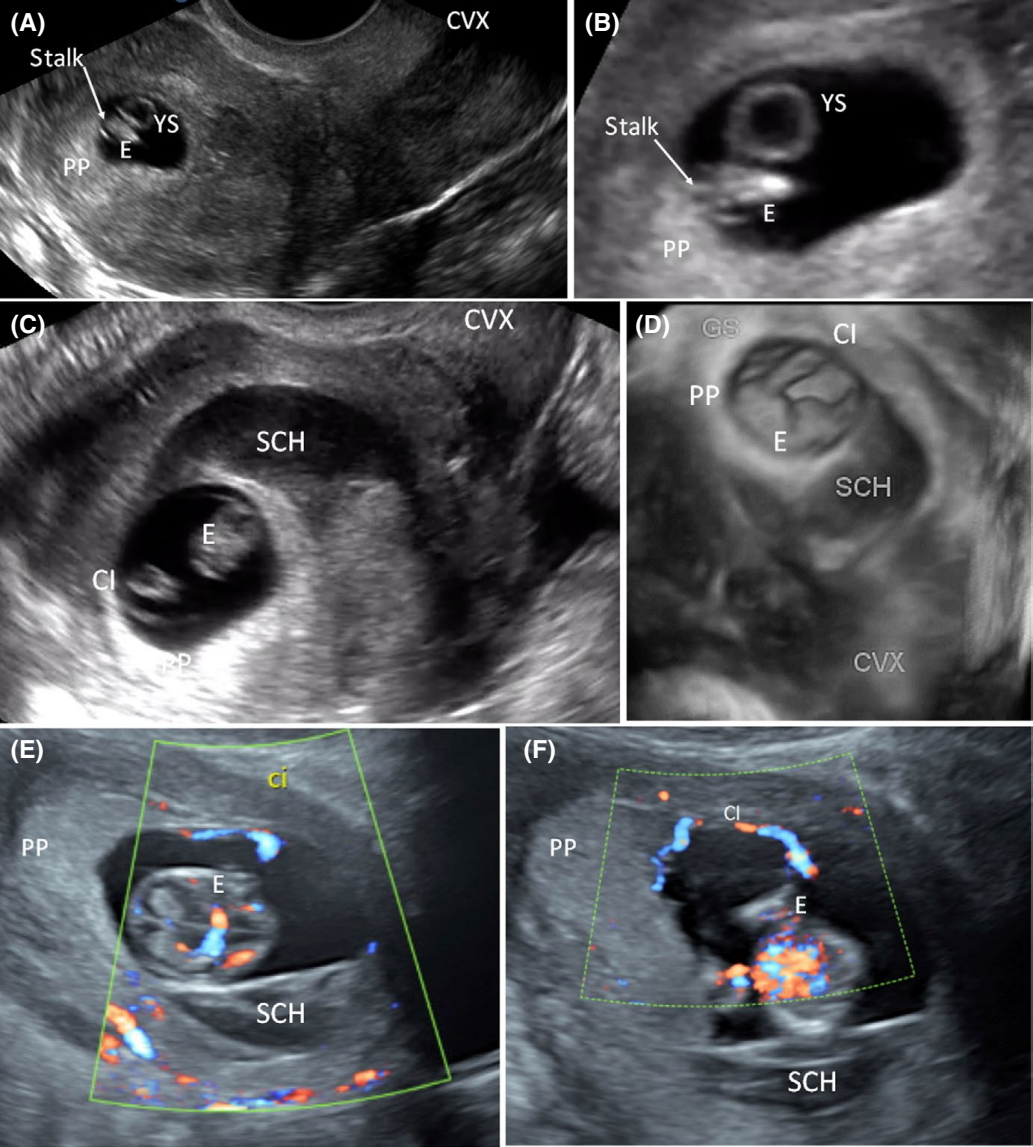
First trimester diagnosis of velamentous cord insertion with ultrasound. A, B, Cord insertion (CI) and placenta location shown at 6 + 4/7 wk of gestation. A, Sagittal view of the uterus with a fundal gestational sac (GS) and anterior‐fundal placenta. B, A close‐up of the embryo (E), the yolk sac (YS), and the embryonal stalk (ES). C, D, CI and placenta location shown at 8 + 3/7 wk of gestation. The placenta was noted to be in fundal‐posterior location at this time. C, Sagittal view of the uterus with a fundal GS and a subchorionic hematoma (SCH) in the lower uterine segment adjacent to the cervix. The marginal/velamentous CI next to the placental (PP) margin was noted. D, 3‐D rendering of the coronal view of the uterus showing the GS, PP, E, CI, and a SCH. E, F, Color Doppler to confirm CI and placenta location. E, Velamentous cord insertion (VCI) at 12 + 6/7 weeks of gestation; F, VCI at 13 + 6/7 weeks of gestation. Abbreviations: CVX, cervix; PP, placenta plate

The patient underwent a fresh single blastocyst transfer after assisted hatching and conceived a singleton intrauterine pregnancy. She was followed with ultrasound every week from 6 to 11 weeks of gestation. Initially, all parameters were normal, including gestational sac (GS) and yolk sac (YS) size, crown‐rump (CRL) length, and heart rate. Placental location was diagnosed as fundal on the initial ultrasound at 6 + 4/7 weeks of gestation (Figure 1A). The patient started experiencing bright red vaginal bleeding at 7 + 2/7 weeks of gestation. A subchorionic hematoma measuring 17 × 9.4 × 15 mm was noted at this time. Mean gestational sac diameter was 18.2 mm, corresponding to the 14th percentile for gestational age. Vaginal bleeding continued, and at 8 + 3/7 weeks of gestation, the subchorionic hematoma enlarged to 21 × 22 × 23 mm. Gestational sac measured 22 mm at this time, corresponding to the 5th percentile for gestational age. A posterior and fundal placenta was again noted, and the subchorionic hematoma appeared to be adjacent to the placental plate. At this time, MCI vs VCI was noted at the anterior‐fundal placental margin on orthogonal views (Figure 1C and D). The finding was confirmed at the following ultrasounds. YS mean diameter was normal throughout the first trimester, measuring 2.3 mm at 6 weeks', 2.6 mm at 7 weeks', 3.1 mm at 8 weeks', 3.9 mm at 9 weeks', and 4.2 mm at 10 weeks' gestations. Cell‐free DNA testing at 10 weeks was consistent with a male fetus and was negative for aneuploidy. However, fetal fraction DNA in the maternal circulation was low, at 3.4%. At 11 + 3/7 weeks of gestation, two subchorionic hematomas measuring 28 × 18 × 12 mm and 26 × 5 × 33 mm, respectively, were noted. At 12 + 6/7 weeks of gestation, stable subchorionic hematomas were noted, in addition to a VCI (Figure 1E). Fetal echogenic bowel was also noted, which was suspected to be secondary to blood ingestion. Interval fetal growth was consistent with gestational age until this time. However, CRL measured 4 days behind at 12 + 6/7 weeks and 8 days behind at 13 + 5/7 weeks. VCI was confirmed at both scans (Figure 1E and F).

At 16 + 5/7 weeks of gestation, the fetus was measuring 13 + 6/7 weeks, by biparietal and head circumference measurements, and there was no cardiac activity. The patient underwent dilation and evacuation and pathology of the products of conception revealed normal findings. Micro‐array chromosome analysis was consistent with a karyotypically normal male fetus.

Placental location can be diagnosed with two‐dimensional ultrasound at 5 to 6 weeks of gestation by evaluating the embryonal stalk (ES) implant in the GS, on both the sagittal (longitudinal) and the transverse views.^6^ This is because the ES will progressively elongate to form the umbilical cord, beneath which the chorion frondosum will develop and become anchored to the opposing basal decidua, thus forming the placenta.^6^ The location of ES in early ultrasound therefore suggests the future locations of the placenta and the PCI, although the placental plate can migrate to adjacent areas, as the pregnancy evolves.^6^ In this particular case, the ES was visible in the antero‐fundal region at 6 weeks, but the placenta was clearly developing in the postero‐fundal region, while the PCI remained in the antero‐fundal region, at 8 weeks, giving us the opportunity to diagnose a marginal/velamentous insertion.

To date, most studies have reported detection of VCI in the second or third trimesters, when certain sonographic features may suggest VCI: the umbilical vessels enter the placental margin parallel to the uterine wall and connect to the superficial placental vessels; the cord insertion is immobile, even when the uterus is shaken; and the umbilical vessels diverge as they traverse the membrane.^7^ In contrast, it is often more difficult to diagnose VCI in the first trimester. Previously, a cord insertion in the lower third of the uterus in the first trimester has been found to be associated with VCI diagnosed later in the pregnancy.^8^

Transection of the exposed umbilical vessels in VCI could be rapidly fatal, as the loss of what appears to be a relatively small amount of blood can be lethal at any gestational age. The overall mean fetal blood volume extrapolated across all three trimesters was 101 mL/kg; at 18 weeks of gestation, the fetal placental blood volume was estimated to be 26 mL.^9^ However, hemorrhage may only be a rare mechanism by which VCI causes pregnancy loss. In fact, acute or chronic cord compression and subsequent placental insufficiency more frequently causes intrauterine growth restriction (IUGR).^1^ In light of the normal fetal karyotype analysis and normal uterine cavity on sonohysterogram, in this case VCI may have been the cause of the rapid IUGR and ultimately the pregnancy loss.

Pregnancy conceived via assisted reproductive techniques is at higher risk of developing MCI or VCI.^3^ It was postulated that IVF procedures per se may be associated with abnormal placentation. In blastocysts, the inner cell mass retains contact with only part of the outer trophectoderm layer or polar region, whereas the remaining tissue that embraces the blastocoelic cavity, so‐called mural region, has no contact with the inner cell mass. The human blastocyst attaches to either the anterior or posterior uterine endometrial surface and appears to do so by its polar rather than the mural region. The IVF micromanipulation procedures, such as ICSI or embryo biopsy, may interfere with the polar trophoblast differentiation and cause an oblique orientation of the blastocyst at the time of nidation.^10^ Since the placenta tends to develop around the implantation site, the oblique orientation may lead to MCI or VCI later on.

In conclusion, VCI may be detected earlier than previously thought, combining the diagnosis of ES implantation site, made at 6 weeks’ gestation, and the position of the placental plate, which becomes more definite at 7‐8 weeks' gestation. Previable pregnancy loss secondary to an abnormal cord insertion may not be preventable, and as the placenta location may evolve as the pregnancy progresses,^6^ it is possible that a low‐lying VCI may resolve later in the pregnancy. However, VCI, regardless of the intrauterine location, is an independent risk factor for multiple chronic and acute obstetric complications. These include vasa previa, which may lead to catastrophic fetal death if left undiagnosed. The prerequisite for timely intervention is early diagnosis. Therefore, early recognition of VCI may impact the survival of an otherwise healthy fetus, once it reaches viability. A diagnosis of VCI early on may inform prenatal surveillance, intrapartum management, and patient counseling. We propose the identification of cord insertion site as part of the first trimester ultrasound surveillance, especially in pregnancies with risk factors of VCI, such as those complicated with first trimester bleeding, twin or higher order pregnancies, or those achieved through assisted reproductive technologies. ^3^

## CONFLICT OF INTEREST

None declared.

## AUTHOR CONTRIBUTIONS

Caiyun Liao MD MPH: served as the primary author of the manuscript draft. Irene Peregrin‐Alvarez MD and Robert Roman MD: collected data and revised the manuscript. Joshua Morris MD: involved in literature review and manuscript revision. Laura Detti MD: involved in conceptualization of the study, data collection, ultrasound examination of the patient and generation of sonographic images, and manuscript revision.

## Data Availability

The authors confirm that the data supporting the findings of this study are available within the article.
